# Investigating the relationship between taste perception of artificial sweeteners and cancer risk

**DOI:** 10.1017/S1368980026101827

**Published:** 2026-01-12

**Authors:** Daisy C. P. Crick, Wenhao Liu, Liang-Dar Hwang

**Affiliations:** 1 Institute for Molecular Bioscience, The University of Queensland, Brisbane, QLD 4067, Australia; 2 Department of Neurology, Peking Union Medical College Hospital, Chinese Academy of Medical Sciences and Peking Union Medical College, Beijing 100730, China

**Keywords:** Artificial sweeteners, Aspartame, Cancer, Mendelian randomisation, UK Biobank, FinnGen

## Abstract

**Objective:**

To investigate whether taste perception of two artificial sweeteners—aspartame and neohesperidin dihydrochalcone (NHDC)—is causally associated with the risk of site-specific cancers.

**Design:**

A two-sample Mendelian randomisation (MR) study.

**Setting:**

Genetic instruments for taste perception (6 for aspartame; 13 for NHDC) were obtained from a genome-wide association study (GWAS) of Australian adolescents, and cancer outcome data were sourced from publicly available GWAS datasets.

**Participants:**

Genetic data for taste perception from 1757 Australian adolescents and genetic data for cancers from large-scale GWAS cohorts, including UK Biobank (*n* 500 000) and FinnGen (*n* 500 000).

**Results:**

A one sd increase in the genetically predicted perceived intensity of NHDC was associated with an increased risk of male genital cancer (OR = 1·11, 95 % CI: 1·04, 1·19) and prostate cancer (OR = 1·03, 95 % CI: 1·01, 1·08) based on FinnGen data. These associations persisted after multivariable MR adjustment for glucose and aspartame perception but were not replicated in the UK Biobank. A weak protective association between aspartame perception and cervical cancer (OR = 0·998, 95 % CI: 0·997, 0·999) was observed, but this attenuated to null in sensitivity analyses.

**Conclusions:**

This study found no compelling evidence that perception of aspartame or NHDC during adolescence causally influences later-life cancer risk. The findings highlight the importance of evaluating individual artificial sweeteners separately in future research examining potential health effects.

In July 2023, the WHO’s International Agency for Research on Cancer classified aspartame, a low-calorie artificial sweetener, as ‘possibly carcinogenic’^(1)^. This classification raised concern because aspartame is widely used as a sugar substitute in food and beverages due to its intense sweetness, approximately 200 times sweeter than sugar, and negligible caloric content^(2)^. Aspartame is a key ingredient in diet soft drinks and is also found in various other products, ranging from dairy items to toothpaste^(3)^.

Observational studies have reported tentative associations between aspartame and cancer occurrence^(4–6)^. A large longitudinal cohort study spanning ten European countries (*n* 477 206) found that daily intake of artificially sweetened beverages was positively associated with hepatocellular carcinoma^(6)^. Similarly, a French longitudinal study (*n* 102 865) reported that higher intakes of aspartame and acesulfame-K (two of the most frequently consumed artificial sweeteners) were associated with increased risks of breast cancer and obesity-related cancers; however, no association was found with prostate cancer^(7)^. In a US study, artificially sweetened drink consumption was associated with liver cancer among participants with diabetes over a 12-year follow-up, but no association was found among participants without diabetes^(4)^. Another prospective cohort study of 934 999 cancer-free participants found a positive association between artificially sweetened beverage intake and pancreatic cancer mortality, after adjustment for confounders including ethnicity, smoking, alcohol use, marital status, education and diet^(5)^.

Although these studies were large, longitudinal and adjusted for possible confounders to mitigate the effects of reverse causation, their observational nature limits causal inference. Residual confounding, measurement error, or selection biases could still underlie the reported associations^(8)^. A meta-analysis of ten case-control studies found no association between artificial sweetener intake and risk for several cancers^(9)^. Further, an independent evaluation by the Joint FAO/WHO Expert Committee on Food Additives) concluded there was no convincing evidence of harm from aspartame, maintaining the current acceptable daily intake of 40 mg/kg body weight^(10)^.

Randomised controlled trials (RCT) are considered the gold standard for establishing causal relationships, but human RCT on artificial consumption and cancer are ethically unfeasible. Consequently, animal models have been used. One RCT in female mice showed that aspartame exposure (administered at doses of 40, 200, or 2500 mg/kg) was associated with increased expression of two oncogenes (c-myc and Ha-ras) and the tumour suppressor p53 in the kidney, bone marrow and lymphoid tissues^(11)^. At 200 mg/kg body weight, elevated gene expression was also observed in the liver, spleen and lungs. A study by Soffritti et al. further reported a positive relationship between aspartame and the incidence of malignant tumours, lymphomas, leukaemias and transitional cell carcinomas in rats, with the effect observed at 20 mg/kg, a dose below the current acceptable daily intake^(12)^. These findings suggest that aspartame may act as a multipotential carcinogenic agent in animal models. However, as with all animal studies, questions remain about the applicability of these findings to human populations^(13)^.

Mendelian randomisation (MR) is an alternative approach for causal inference where RCT are not feasible^(14,15)^. MR uses genetic variants as instrumental variables to proxy exposures of interest, exploiting the random allocation of alleles at conception to reduce confounding and reverse causation^(16)^. Two-sample MR approaches can estimate causal effects by using genetic variant effects on the exposure in one study and genetic effects on the outcome in a separate study. Thus, statistically powerful MR studies investigating diet-health relationships can be conducted in large biobanks. Furthermore, the causal effects estimated in MR represent *long-term effects*, providing considerable practical advantages over short-term, expensive RCT.

Recent MR studies have explored the association between artificial sweetener intake and cancer risk. While several studies report evidence suggestive of a causal relationship—particularly for obesity-related cancers^(17)^, respiratory cancers^(18)^, breast and liver cancers^(19)^, and cancer-related mortality^(20)^—others have found no strong evidence for associations with specific outcomes such as colorectal cancer^(21)^. However, these studies have typically treated ‘artificial sweeteners’ as a single, broad exposure, without differentiating between individual compounds, which limits the ability to draw conclusions about the health effects of specific sweeteners like aspartame or NHDC.

In this study, we use MR to examine the relationship between the genetic predisposition to the perception of two artificial sweeteners—aspartame and neohesperidin dihydrochalcone (NHDC)—and cancer risk. Taste is a key determinant of food choices^(22)^, so differences in how people experience sweetness could affect how much of these sweeteners they consume. While aspartame has been the focus of intense scrutiny, NHDC—a citrus-derived compound approved for use as a sweetener in countries of the European Union^(23)^—has received comparatively little epidemiological attention. Despite its distinct chemical structure and prolonged sweetness profile, its functional similarity to aspartame makes it a relevant candidate for study in parallel. By focusing on the genetic basis of sweetener perception, this study aims to provide new evidence regarding the potential causal link between artificial sweetener exposure and cancer, thereby informing the interpretation of recent public health guidance and supporting future risk assessments of both commonly used and lesser-studied sweeteners.

## Methods

This study was conducted following the STROBE-MR guideline^(24)^.

### Study design and data sources

We used summary-level data from a genome-wide association study (GWAS) of the perceived intensities of aspartame and NHDC^(25)^ performed in an Australian adolescent twin sample (*n* 1757, age 12–25 mean, 51·2 % female). This cohort represents the only GWAS with available data on the perception of aspartame or NHDC. Taste tests were conducted between 2003 and 2014. Participants were asked to taste 4 sweet (0·60 M glucose, 0·30 M fructose, 8·0 × 10^–5^ M neohesperidin dihydrochalcone, NHDC and 1·4 × 10^–3^ M aspartame) and 5 bitter (6·0 × 10^–4^ M propylthiouracil, 2·0 × 10^–4^ M sucrose octaacetate, 1·81 × 10^–4^ M quinine, 0·05 M caffeine and 4·99 × 10^–6^ M denatonium benzoate) solutions and water and rate their perceived intensity ratings using a generalised labelled magnitude scale, with labels of no sensation (0 mm), barely detectable (2 mm), weak (7 mm), moderate (20 mm), strong (40 mm), very strong (61 mm) and strongest imaginable (114 mm). The GWAS was performed using the z-score of the square-root-transformed perceived intensities.

Outcomes of cancers included in this study were based on their availability in the MRC IEU database (https://gwas.mrcieu.ac.uk), resulting in 21 site-specific cancers as follows: skin, male genital, prostate, cervical, colon, ovarian, oral cavity, bowel, rectal, bronchial, endometrial, non-small cell lung, pharyngeal, breast, pancreatic, ureteral, endocrine, head and neck, haematological, bladder and kidney cancer. However, in some cases, the exposure instrumental variables (or their correlated genetic variants) were unavailable in the cancer GWAS. Where possible, we replicated our analyses using site-specific cancer data from Neale lab, which were GWAS performed in the UK Biobank (http://www.nealelab.is/uk-biobank/).

When there was evidence of an association using either database, we further validated the results using GWAS from other sources. To minimise potential bias, we ensured that there was no overlap between individuals who participated in the GWAS of perceived intensity of artificial sweeteners and those who participated in the cancer GWAS. Only GWAS of European ancestry were included in the study to minimise bias due to population stratification. See online supplementary material, Supplemental Table 1 for information on the GWAS used in these analyses.

### Instrument selection

We clumped GWAS summary results statistics of the perception of aspartame and NHDC based on their linkage disequilibrium (window size = 10 000 kb; r2 threshold = 0·001; significant threshold for index SNP = 1 × 10^–5^). We identified 6 SNP available as instrumental variables for the perceived intensity of aspartame and 13 SNP available for the perceived intensity of NHDC. None of these SNP were palindromic (i.e. A/T or C/G), excluding the possibility of incorrect effect-allele assignment between exposure and outcome datasets. SNP were then harmonised with the cancer GWAS. SNP harmonised with cancer GWAS identified through the IEU Open GWAS and Neale lab are presented in online supplementary material, Supplemental Tables 2 and 3, respectively.

### Two-sample Mendelian randomisation

The MR inverse-variance weighted (IVW) random-effects model^(26)^ was used to estimate effects in the primary analysis. We used the false discovery rate (FDR) correction to account for multiple testing, and sensitivity analyses were performed on associations that had an FDR-adjusted *P*-value < 0·05. Given that the sensitivity methods (simple mode, MR-Egger, weighted median and weighted mode) make different assumptions about pleiotropy, consistency between the methods strengthens causal inference (see online supplementary material, Supplemental Table 4 for the assumption of each MR method). Given that the exposure was the z-score of the square-root-transformed perceived intensity of artificial sweeteners, the causal effect can be interpreted as a change in the odds of cancer risk per sd increase in the genetically predicted perceived intensity, approximated as a change in the perception scale from moderate/weak to strong/weak.

There are three core assumptions of MR: (i) The relevance assumption which describes how genetic variants must be robustly associated with the exposure, (ii) the independence assumption where genetic variants should not be associated with the outcome through confounding, and (iii) the exclusion restriction assumption which states that genetic variants should influence the outcome solely through their effect on the exposure (online supplementary material, Supplemental Figure 1).

To address the first assumption, we calculated F-statistics for each SNP using the formula F = *β* ^2/^SE^2^, where *β* is the SNP effect estimate and se is the standard error of *β*. F-statistics > 10 suggest that a SNP is strongly associated with the exposure. Therefore, although we used a more relaxed threshold of *P* < 1 × 10^–5^ (compared to the traditional GWAS significant threshold of *P* < 5 × 10^–8^) to identify appropriate SNP, we showed that all identified SNP had F-statistics > 10. We also calculated the proportion of variance in phenotype explained by each SNP^(27)^. F-statistics and the proportion of variance explained are provided in online supplementary material, Supplemental Table 2.

While the other two assumptions cannot be statistically investigated, we performed several statistical tests to interrogate them. For example, we used Steiger filtering to confirm that SNP included in the analyses explained more variance in the exposure than in the outcome. This mitigates the possibility of selecting reverse causal variants (i.e. SNP are associated with the exposure through the outcome)^(28)^. Further divergent results between sensitivity analyses such as MR-Egger, weighted median, weighted mode and MR pleiotropy residual sum and outlier test suggest that the third MR assumption may be violated^(29–32)^. We investigated pleiotropy using the MR-Egger intercept^(33)^ and MR pleiotropy residual sum and outlier global test^(32)^ and heterogeneity among instrumental variables using Cochrane’s Q. We also used the leave-one-out analyses^(34)^ to assess whether MR estimates were driven by a particular SNP with a strong effect.

### Multivariable Mendelian randomisation

Multivariable MR (MVMR) is an extension of MR that estimates the *direct* independent effect of exposures on an outcome instead of the *total* effect of the exposures on the outcome^(35)^. It can also be used to account for pleiotropic pathways from an instrument to the outcome, i.e. the third MR assumption.

We first investigated the direct causal effect of aspartame and NHDC taste perception on cancers, independent of each other and the perceived sweetness intensity of glucose, a natural sweetener. This is because the perception of artificial sweeteners and sugars are correlated^(36)^. By using MVMR, we can ensure that the effects of artificial sweeteners on cancer risk we report are not biased by the perception of other sweeteners, be they natural or artificial. Following the same instrument selection procedure as in the univariable analysis, we identified 7 SNP associated with the perceived sweetness intensity of glucose^(37)^. A total of 25 SNP (6 for aspartame, 13 for NHDC and 7 for glucose) were used to perform the MVMR.

**Figure 1.**
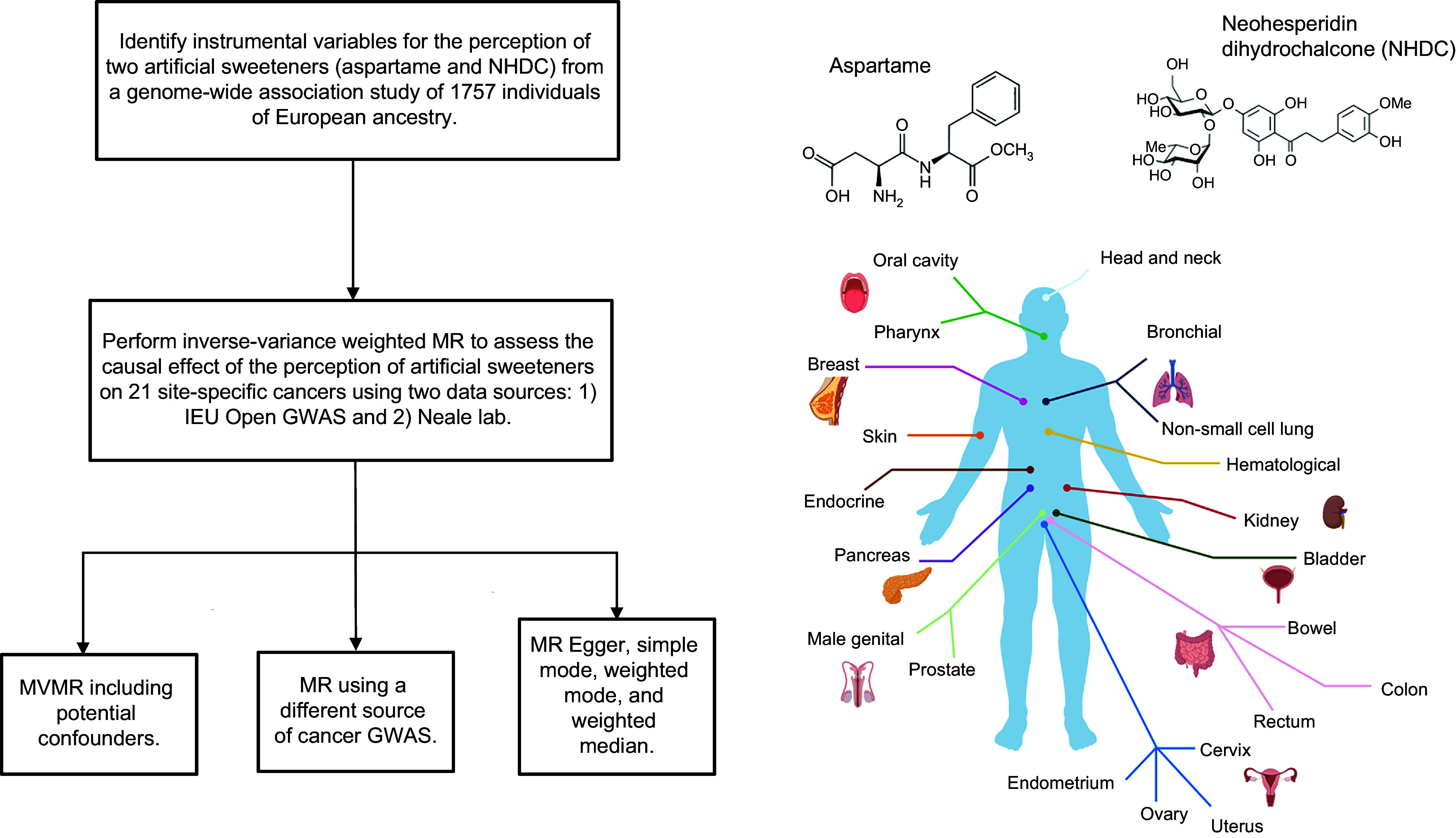
Overview of the analyses performed in the present study.

An overview of the analyses is shown in Figure 1.

### Software

All computational analyses were executed using R software (version 4.2.2) and the following R packages: TwoSampleMR (version 0.5.7), MR-PRESSO (version 1.0) and MVMR (version 0.4).

## Results

### MR analysis of NHDC perception and cancer risk

**Figure 2.**
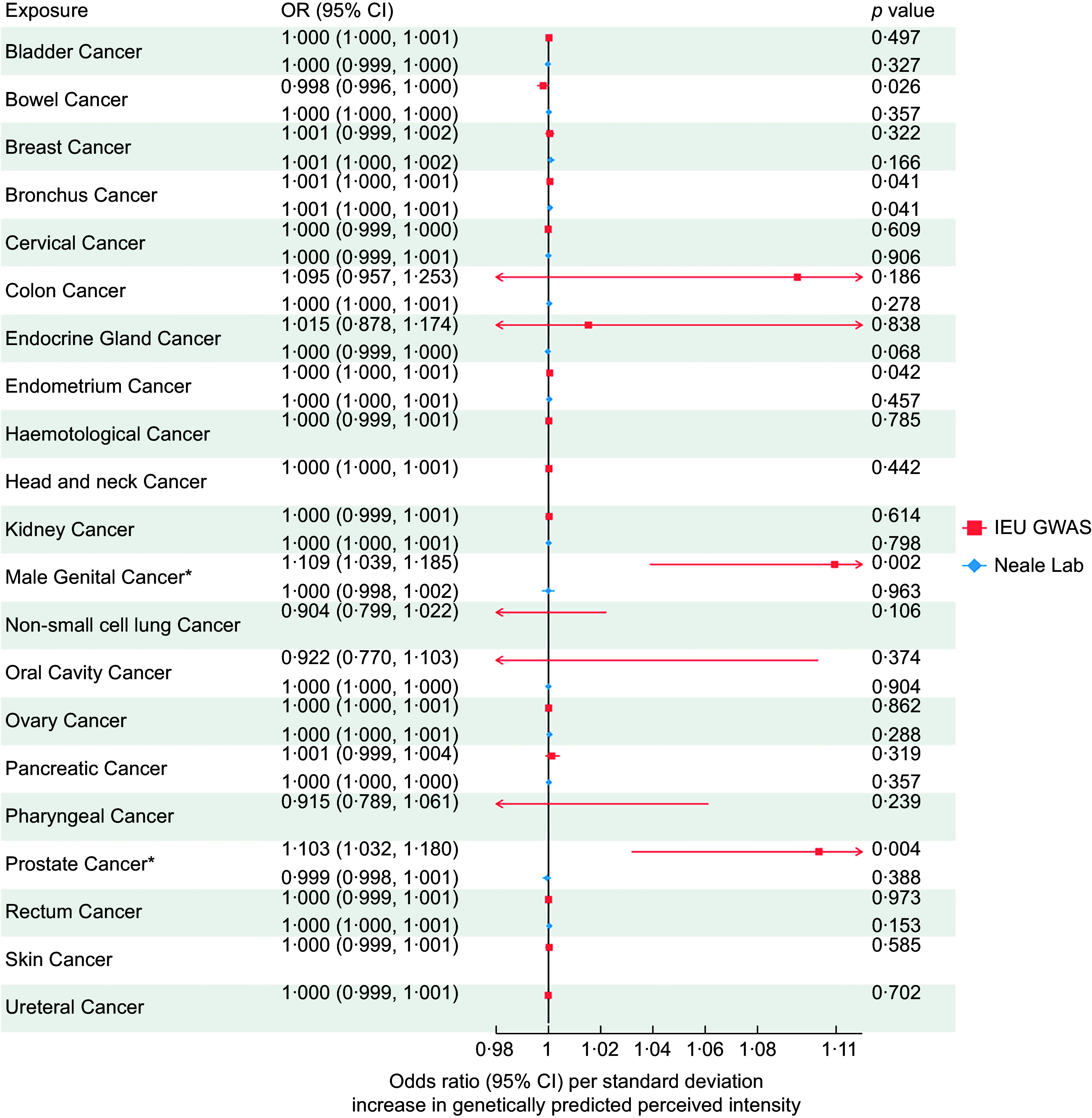
Inverse-variance weighted Mendelian randomisation results for the association between neohesperidin dihydrochalcone perception and 21 site-specific cancers using genome-wide association studies (GWAS) data of cancers from IEU GWAS and Neale Lab.

**Figure 3.**
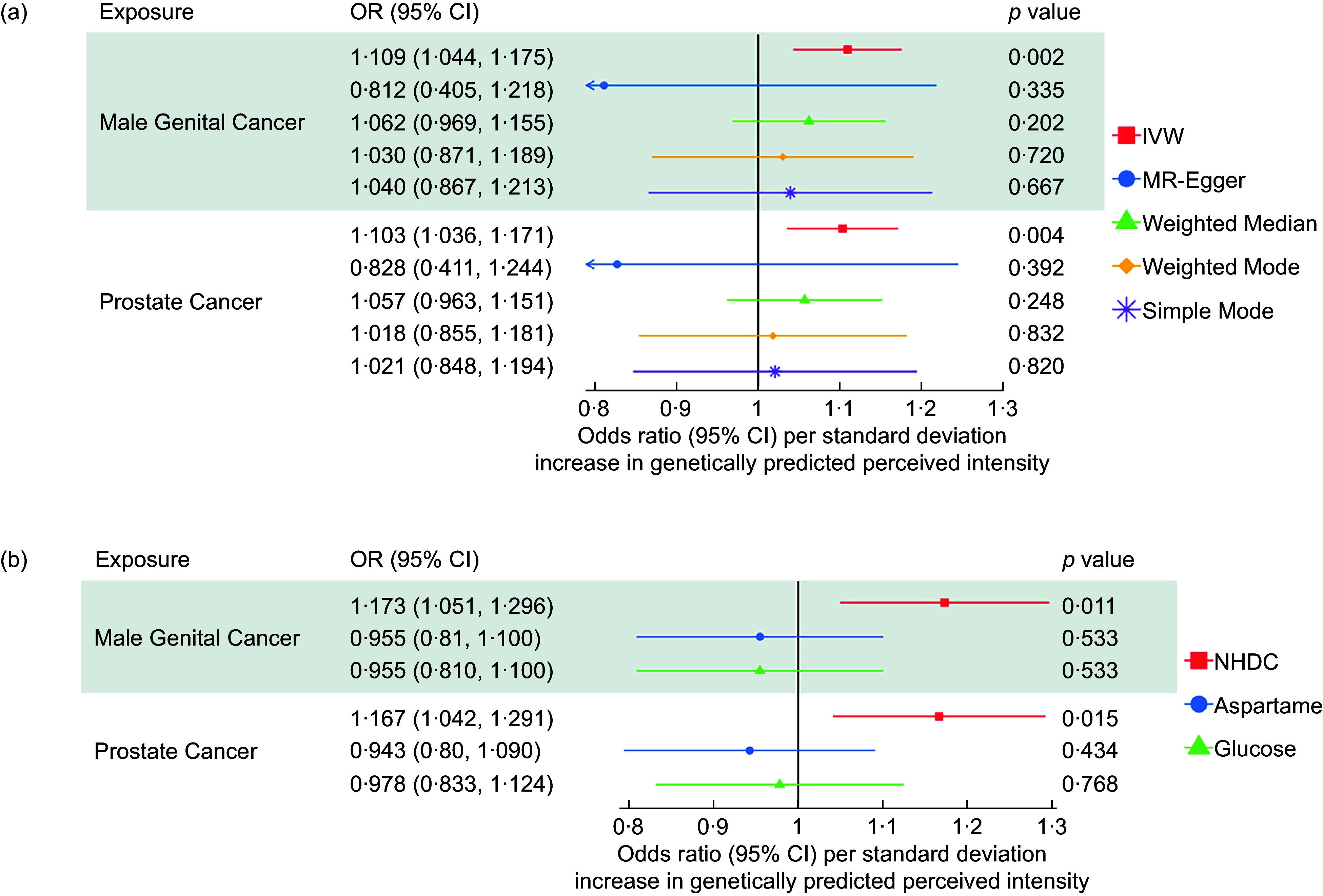
Sensitivity analyses for the causal association between neohesperidin dihydrochalcone and prostate cancer and male genital cancer using data from the IEU open GWAS.

Across all 21 site-specific cancers, we found evidence that the increased perceived intensity of NHDC increased the risk of bowel cancer, bronchial cancer, endometrial cancer, male genital cancer and prostate cancer. IVW-MR results for all analysed cancers are presented in Figure 2. However, after correction for multiple testing, only male genital cancer (IVW OR: 1·109, 95 % CI: 1·039, 1·185) and prostate cancer (OR: 1·03, 95 % CI: 1·032, 1·80) passed the FDR-adjusted *P*-value, and thus, only they were taken forward for further analyses. Associations attenuated to the null in the sensitivity analyses (MR-Egger, Weighted Median, Weighted Mode and Simple Mode); however, the direction of effects was primarily consistent (Figure 3).

In the MR replication analysis, using cancer data from Neale lab (i.e. UK Biobank), we found no evidence for a causal effect of NHDC on prostate cancer (OR: 1·00, 95 % CI: 0·998, 1·001) or male genital cancer (OR: 1·00, 95 % CI: 0·998, 1·003). Further, when using a GWAS meta-analysis of prostate cancer, including data from both FinnGen and UK Biobank^(37)^, we found no evidence of a causal effect (OR: 1·00, 95 % CI: 0·999, 1·001). The harmonised SNP used in this MR analysis are presented in online supplementary material, Supplemental Table 5.

Heterogeneity was not detected, and the MR-Egger intercept test indicated the absence of pleiotropic effects (see online supplementary material, Supplemental Table 6). MR pleiotropy residual sum and outlier global test detected no presence of pleiotropy in the association with male genital cancer or prostate cancer. Null associations with other cancers remained after removal of the outliers (see online supplementary material, Supplemental Table 7).

Results from the leave-one-out analysis suggested that the IVW-MR association between NHDC and prostate cancer and NHDC and male genital cancer observed in our study were unlikely to be influenced by any one extreme SNP. (see online supplementary material, Supplemental Figures 2 and 3)

The MVMR results suggest that when accounting for glucose perception and aspartame perception, the effect of NHDC on the risk of male genital cancer and prostate cancer remained (Figure 3).

### MR analysis of aspartame perception and cancer risk

**Figure 4.**
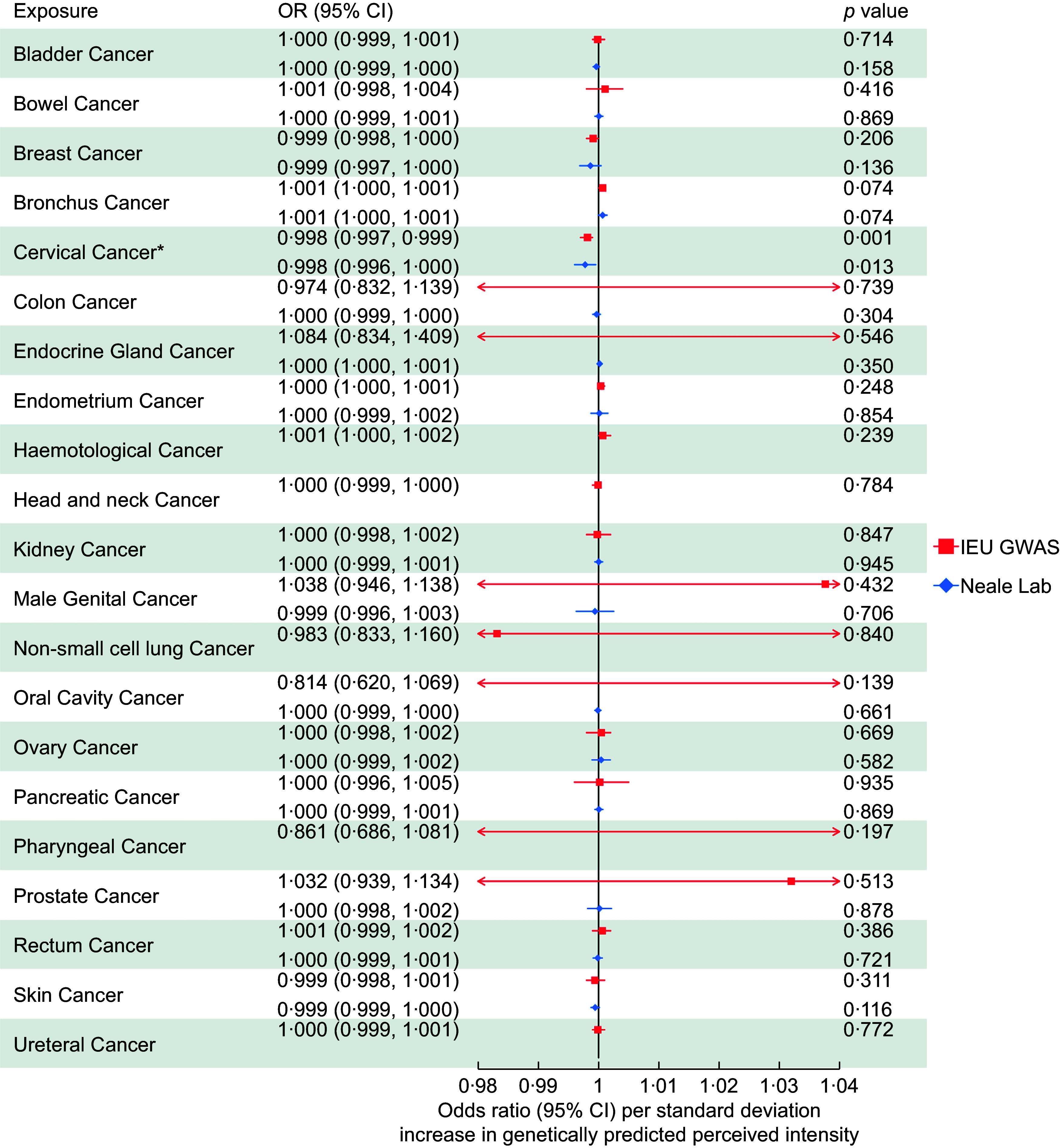
Inverse-variance weighted Mendelian randomisation results for the association between aspartame perception and 21 site-specific cancers using genome-wide association studies (GWAS) data of cancers from IEU GWAS and Neale Lab.

Across all 21 site-specific cancers, we found evidence that an increased perceived intensity of aspartame reduced the risk of cervical cancer (OR: 0·998, 95 % CI: 0·997, 0·999). IVW-MR results for all cancers are presented in Figure 4.

**Figure 5.**
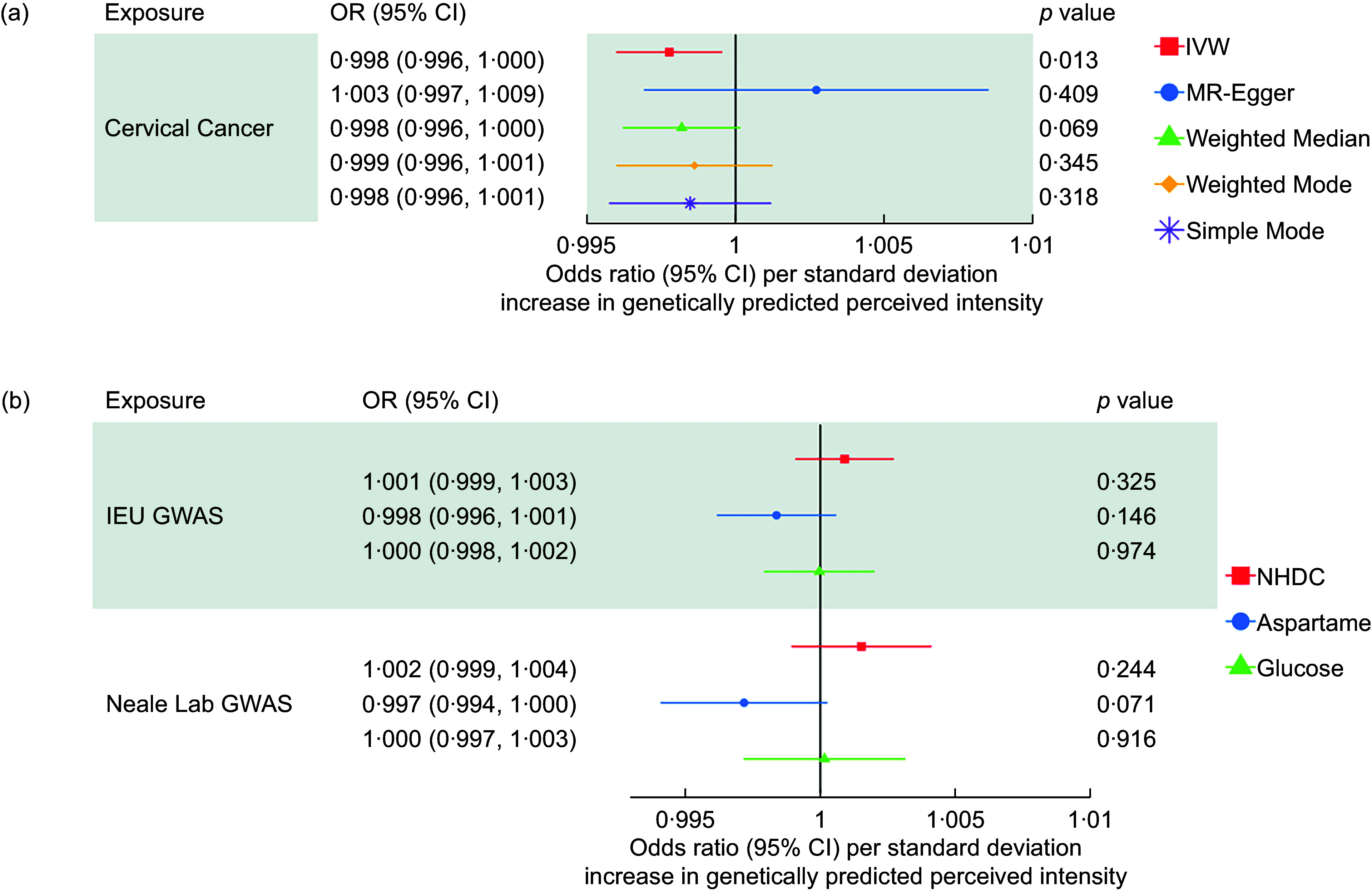
Sensitivity analyses for the causal association between aspartame and cervical cancer using data from the IEU open GWAS.

We were unable to perform sensitivity analyses for the association with cervical cancer using data from the IEU Open GWAS dataset due to only two SNP being available (i.e. rs77739585 and rs855749). However, the replication MR using GWAS results from Neale lab also suggested that aspartame has a protective effect on cervical cancer risk (OR: 0·998, 95 % CI: 0·996, 1·000). Results attenuated to the null across the sensitivity analyses, although the direction of effect was largely consistent (Figure 5).

We performed a further follow-up analysis using a GWAS meta-analysis of cervical cancer^(38)^, including data from FinnGen, UK Biobank, Kaiser Permanente and Estonia Biobank. We replicated the direction of effect; however, the 95 % CIs overlap the null (OR: 0·937, 95 % CI: 0·866, 1·008). The harmonised SNP used in this MR analysis are presented in online supplementary material, Supplemental Table 8.

Heterogeneity was not detected, and the MR-Egger intercept test indicated the absence of pleiotropic effects (see online supplementary material, Supplemental Table 5). Results from the leave-one-out analysis suggested that the association between aspartame and cervical observed in our study was unlikely to be influenced by any one extreme SNP (see online supplementary material, Supplemental Figure 4).

The protective effect of the perceived intensity of aspartame on cervical cancer attenuated to the null in the MVMR analysis after adjusting for the effects of NHDC and glucose. This occurred when using the cervical cancer GWAS from both the IEU Open GWAS and the GWAS from Neale lab (Figure 5).

## Discussion

In this study, we used MR to investigate the potential causal relationship between taste perception of artificial sweeteners and the risk of 21 site-specific cancers. Overall, we found no compelling evidence to support a causal role of the perceived intensity of aspartame or NHDC in cancer risk. While suggestive associations were observed between increased perceived intensity of NHDC and higher risk of male genital cancer and prostate cancer, as well as between aspartame perception and a reduced risk of cervical cancer, these associations attenuated to null in sensitivity analyses using alternative datasets or analytical models.

The associations for NHDC were observed when using cancer GWAS data from FinnGen but were not replicated in independent datasets such as the UK Biobank or meta-analyses that included both UK and Finnish populations. This lack of replication raises uncertainty about the robustness of the observed associations and suggests the possibility of population-specific effects, potentially driven by differences in consumption patterns, genetic background or food regulations. While both the UK and Finland follow Food Safety Authority guidelines, including the 2022 increase in NHDC’s acceptable daily intake from 5 to 20 mg/kg body weight^(39)^, regional variation in product availability, marketing or consumer habits may still play a role. The persistence of the NHDC signal in MVMR models adjusting for aspartame and glucose perception suggests that this effect, if real, may be independent of general sweet taste perception. However, given the lack of replication of this association in the UK Biobank, these findings should be interpreted with caution and warrant further investigation.

For aspartame, we found weak evidence of a protective association with cervical cancer, which was directionally consistent across multiple datasets but attenuated to the null in sensitivity and multivariable analyses. One potential explanation is that individuals with heightened aspartame perception may be more aware of the presence of aspartame in food and, due to negative public perceptions^(40)^, may choose to avoid products containing it, thereby reducing their actual intake. However, this remains speculative, and the attenuation in MVMR suggests possible confounding by perception of other sweeteners, such as NHDC or glucose.

Importantly, our findings do not support the WHO’s concern about a potential link between aspartame and kidney cancer, as we observed no association between the perception of either sweetener and kidney or bladder cancers. This discrepancy may arise because observational findings in prior reports are susceptible to confounding, particularly by factors such as obesity, which itself increases cancer risk and is associated with higher consumption of low-calorie sweeteners^(41–45)^. MR addresses such confounding and reverse causality, which may explain the lack of association in our study.

However, some limitations must be acknowledged. First, the instrumental variables used in our MR analyses were based on perceived intensity of artificial sweeteners rather than actual preference or consumption, which may only partly capture real-world exposure. While previous research suggests that perceived intensity is positively correlated with preference (*r* = 0·21 for 12 % sucrose)^(46)^, this relationship may not hold across all sweeteners or populations. Further work is needed to identify genetic instruments for sweetener preference or intake.

Second, we used a less stringent *P*-value threshold (*P* < 1 × 10^-5^) for SNP selection to increase the number of instruments and the proportion of variance explained in exospores. Although all instruments had F-statistics > 10, relaxing this threshold may have introduced weaker or potentially invalid instrumental variables, possibly biasing estimates towards the null in a two-sample MR framework.

Third, while restricting analyses to GWAS of European ancestry minimised potential bias from population stratification, it limits the generalisability of our findings to non-European populations. In addition, as the taste perception phenotypes were measured in adolescents, it remains uncertain whether these associations are age-specific or can be generalised to adults.

Finally, our findings suggest a possible, weak but consistent effect of NHDC perception on male genital and prostate cancers in one population. This was supported by directionally consistent results across sensitivity analyses and MVMR, though not confirmed in replication datasets. Further research using genetic instruments for sweetener consumption, ideally in large, diverse cohorts, will be essential to disentangle whether these associations are genuine or artefactual.

### Conclusion

In summary, we found no robust evidence for a causal relationship between the perception of artificial sweeteners—aspartame and NHDC—and the risk of 21 site-specific cancers. Although our findings contribute to the ongoing discourse surrounding artificial sweeteners and cancer risk, the non-replication across datasets highlights the need for cautious interpretations. As global consumption of artificial sweeteners continues to rise^(47)^, and public concern about their safety persists, further research is warranted to clarify whether and how sweetener perception influences consumption and long-term health outcomes. Future studies incorporating genetic instruments for sweetener preference and intake, alongside cross-country comparisons and richer phenotyping, will be essential to build a more comprehensive understanding of the potential role of artificial sweeteners in cancer risk.

## Supporting information

Crick et al. supplementary material 1 Crick et al. supplementary material

Crick et al. supplementary material 2 Crick et al. supplementary material

Crick et al. supplementary material 3 Crick et al. supplementary material

## Supplementary material

For supplementary material accompanying this paper visit https://doi.org/10.1017/S1368980026101827.
