# Supplementary material for: Investigating the relationship between taste perception of artificial sweeteners and cancer risk
Source: Public Health Nutr. 2026 Jan 12;29(1):e18. doi: 10.1017/S1368980026101827 (PMC12895476; doi:10.1017/S1368980026101827)
Supplement: Crick et al. supplementary material 2 — Crick et al. supplementary material [file S1368980026101827sup002.pdf]

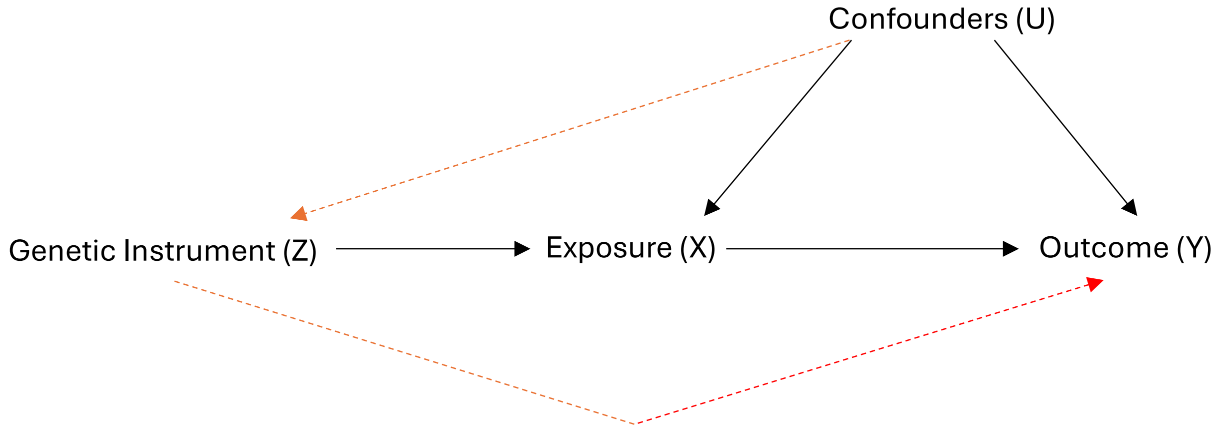

Supplementary Figure 1: Pictural representation of the basic principles of Mendelian randomization. The exposure (X) is causally associated with the outcome (Y) if the three assumptions hold. 1) the genetic variant (Z) is associated with X; 2) Z is independent of any measured or unmeasured confounding factors and; 3) there is no association between Z and Y except through X. The red lines indicate potential violations of MR assumptions and must not be present for Z to be a valid instrumental variable.

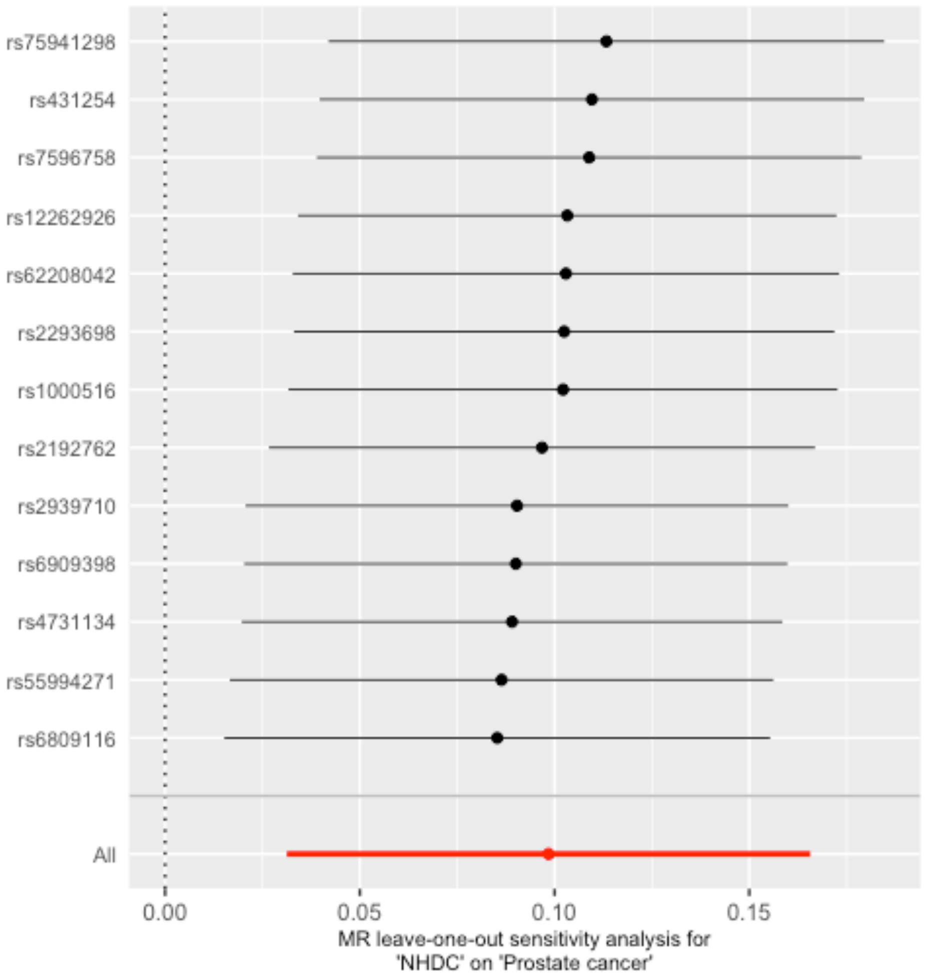

Supplementary Figure 2: Leave-one-out analysis for the IVW analysis investigating the causal effect of NHDC on Prostate cancer.

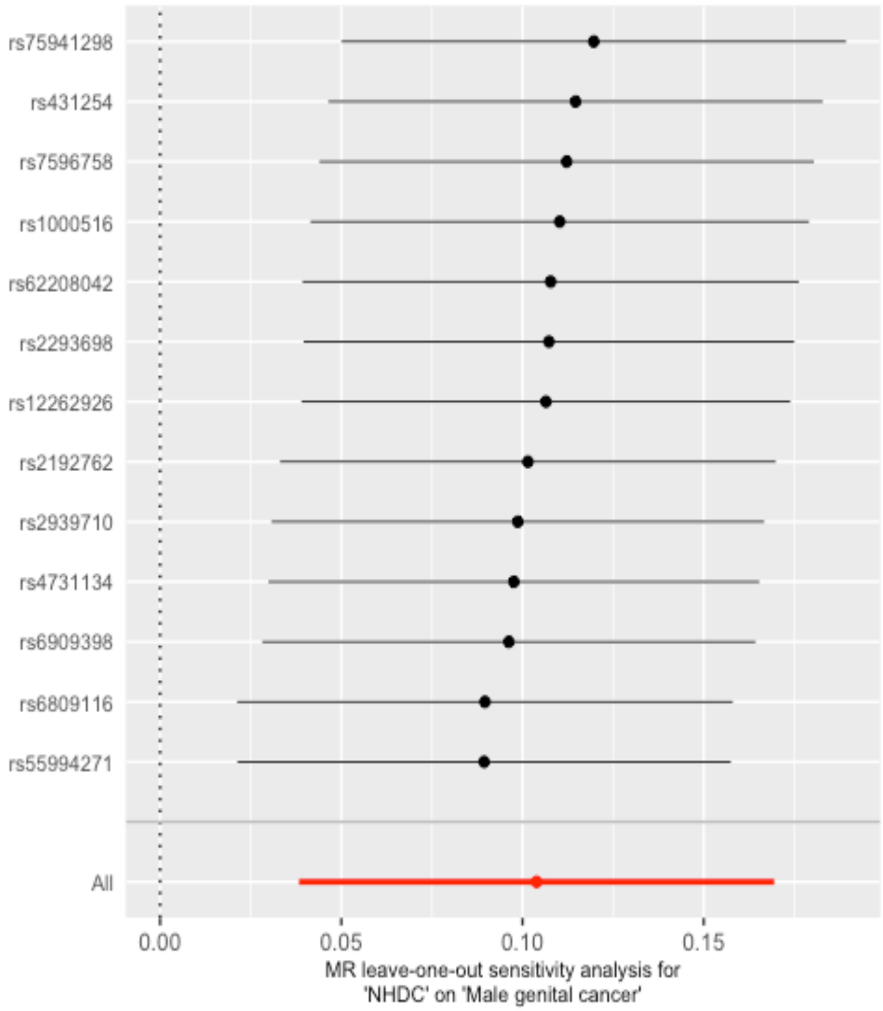

Supplementary Figure 3: Leave-one-out analysis for the IVW analysis investigating the causal effect of NHDC on male genital cancer.

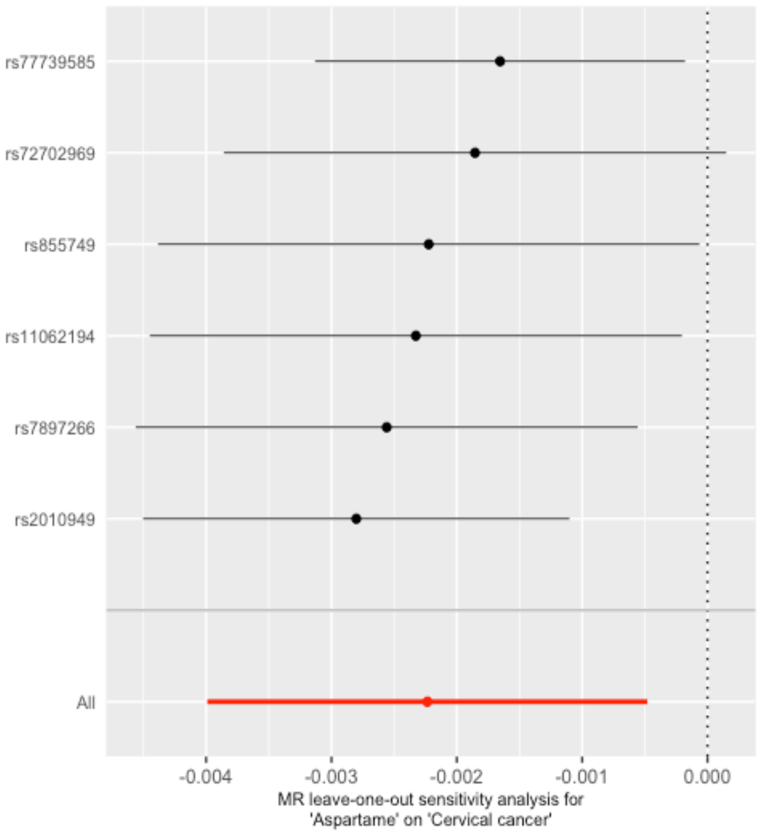

Supplementary Figure 4: Leave-one-out analysis for the IVW analysis investigating the causal effect of aspartame on cervical cancer.
